# Radiodynamic Therapy with Acridine Orange Is an Effective Treatment for Bone Metastases

**DOI:** 10.3390/biomedicines10081904

**Published:** 2022-08-05

**Authors:** Gemma Di Pompo, Katsuyuki Kusuzaki, Marco Ponzetti, Vito Ferdinando Leone, Nicola Baldini, Sofia Avnet

**Affiliations:** 1 Biomedical Science and Technologies and Nanobiotechnology Laboratory, IRCCS Istituto Ortopedico Rizzoli, 40136 Bologna, Italy; 2 Department of Musculoskeletal Oncology, Takai Hospital, Tenri 632-0372, Japan; 3 Department of Biotechnological and Applied Clinical Sciences, University of L’Aquila, 67100 L’Aquila, Italy; 4 Centro Oncologico Veterinario, 40037 Bologna, Italy; 5 Department of Biomedical and Neuromotor Sciences, University of Bologna, 40126 Bologna, Italy

**Keywords:** radiodynamic therapy, acridine orange, tumor acidosis, bone metastases, tumor microenvironment

## Abstract

Current multimodal treatment of bone metastases is partially effective and often associated with side effects, and novel therapeutic options are needed. Acridine orange is a photosensitizing molecule that accumulates in acidic compartments. After photo- or radiodynamic activation (AO-PDT or AO-RDT), acridine orange can induce lysosomal-mediated cell death, and we explored AO-RDT as an acid-targeted anticancer therapy for bone metastases. We used osteotropic carcinoma cells and human osteoclasts to assess the extracellular acidification and invasiveness of cancer cells, acridine orange uptake and lysosomal pH/stability, and the AO-RDT cytotoxicity in vitro. We then used a xenograft model of bone metastasis to compare AO-RDT to another antiacid therapeutic strategy (omeprazole). Carcinoma cells showed extracellular acidification activity and tumor-derived acidosis enhanced cancer invasiveness. Furthermore, cancer cells accumulated acridine orange more than osteoclasts and were more sensitive to lysosomal death. In vivo, omeprazole did not reduce osteolysis, whereas AO-RDT promoted cancer cell necrosis and inhibited tumor-induced bone resorption, without affecting osteoclasts. In conclusion, AO-RDT was selectively toxic only for carcinoma cells and effective to impair both tumor expansion in bone and tumor-associated osteolysis. We therefore suggest the use of AO-RDT, in combination with the standard antiresorptive therapies, to reduce disease burden in bone metastasis.

## 1. Introduction

Bone metastases (BM) are frequent and devastating events in many cancer types, including breast, prostate, lung, kidney, and thyroid cancer, among others [1]. The current standard care for patients with BM aims to treat the associated skeletal-related events, such as fracture, spinal cord compression, pain, and hypercalcemia [1]. It is based on drug administration, including bisphosphonates, inhibitors of receptor activator of nuclear factor-kappa b ligand (RANKL), radiopharmaceuticals, and analgesics, as well as on local approaches, such as radiotherapy, vertebroplasty or other surgical procedures, and nerve decompression. However, these multimodal strategies are often associated with serious side effects and only partially result in tumor control [2], emphasizing the need for novel therapeutic options. These should be more specific and focused on the mechanisms behind tumor progression in bone.

Osteolytic BM follow the establishment of a vicious cycle that simultaneously favors tumor growth and bone destruction [3]. To adapt and survive in the premetastatic niche, cancer cells interfere with the regular cycle of bone remodeling by stimulating the aberrant recruitment and activation of osteoclasts (OCs) [4], the cells that are responsible for bone resorption even under physiological conditions. This vicious cycle is coupled with the establishment of an acidic microenvironment that contributes to local tumor aggressiveness [5]. Acidosis is a hallmark of cancer that increases tumor invasiveness [6] and chemoresistance [7]. In BM, extracellular acidosis results from both the altered cancer cell metabolism (the so-called Warburg effect [8]) and the active bone resorption by OCs [5,9]. Notably, in BM, the extracellular acidosis can directly foster osteolysis, both by degrading the mineral component of bone and by favoring the post-translational modification of collagenases, such as the matrix metalloproteinases (MMPs) [10,11,12]. Most recently, we have demonstrated that acidosis triggers osteolysis also by inducing the secretion of inflammatory and pro-osteoclastogenic mediators, such as interleukin 6 and 8 (IL-6 and IL-8) or tumor necrosis factor (TNF), in osteoblasts, the bone-forming cells [13]. Finally, in BM, tumor acidosis is also responsible for pain induction via the direct stimulation of ion-sensitive nociceptors on the cell membrane of sensory neurons, and by promoting hyperalgesia through the induction of the release of neuromodulators (e.g., the brain-derived neurotrophic factor (BDNF), IL-6, IL-8) in tumor-associated mesenchymal stromal cells [9,14]. For all these reasons, tumor acidosis represents a valuable therapeutic target for BM.

Acridine orange (AO) is a fluorescent and weakly basic molecule that accumulates in the acidic cell compartments and in acidifying cells. Its protonation causes the formation of di- and oligomeric aggregates that appear as bright orange fluorescent granules after blue-light excitation [15,16]. AO easily penetrates the cell membranes due to its low molecular weight (265.36 g/mol). Inside the cell, it concentrates within lysosomes and other acidic organelles. Accumulated AO can also be activated and not only excited by blue light [17]. Once activated, AO gives origin to activated oxygen from intracytoplasmic oxygen. Singlet oxygen, in turn, oxidizes the fatty acids of lysosomal membranes, leading to the leakage of lysosomal enzymes such as proteases, lipases, and nucleases into the cytosolic space [17,18,19]. Consequently, the lysosome membrane is severely permeabilized (lysosomal membrane permeabilization (LMP)). This property of AO can be advantageously used to assess the lysosomal stability in living cells; the rate at which AO freely diffuses from the lysosomes into the cytosol, after lysosomal disruption, can be easily quantified. Such a type of approach has been already used to evaluate susceptibility to lysosomal pharmacological targeting since lysosomes are essential regulators of nutrient homeostasis, apoptosis, autophagy, and membrane trafficking, especially in cancer cells, and are also involved in chemoresistance [20,21,22]. Notably, highly glycolytic cancer cells actively reduce the excess of intracellular protons through their compartmentalization into lysosomes that are, therefore, more acidic and larger than in normal cells [23,24]. As a result of the active transport across the plasma membrane and storage within the lysosomal compartment of the excess of protons, cancer cells have a reversed pH gradient consisting of a higher intracellular pH and a lower extracellular and lysosomal pH [25].

The ability of AO to selectively accumulate in tumor lysosomes and, if activated, to induce LMP may be useful not only for the set-up of an in vitro lysosomal stability assay but also to treat cancer, since, depending on the rate of LMP, it can eventually culminate in uncontrolled necrosis or activation of apoptotic pathways [26]. Indeed, the tumor-selective tropism of AO has been confirmed in several malignancies and has been successfully used to detect tumor margins during surgery and excise the tumor mass under a fluorescence microscope, with minimal damage to the surrounding healthy tissues [27,28,29,30,31] in sarcomas as well as in breast, skin, conjunctival, renal, lung, and liver carcinoma [27,32,33,34,35,36]. In addition, while AO accumulation and activation by photon energy from visible light (photodynamic therapy (AO-PDT)) or X-rays (radiodynamic therapy (AO-RDT)) has been proved in osteosarcoma and rhabdomyosarcoma [27,37,38,39], it has never been explored in BM.

In BM, lysosomes are also very important organelles in OCs, since acidification of the resorption lacunae and related bone resorption depends on lysosome pH [40]. Therefore, in BM, AO treatment might lead to its intracellular accumulation and excitation both in OC and tumor cells, as they both have very acidic lysosomes.

In this study, for the first time, we investigated two different anticancer strategies that can target tumor acidification, and focused on AO-RDT, by using both in vitro and in vivo models of BM.

## 2. Materials and Methods

### 2.1. Cell Cultures

#### 2.1.1. Carcinoma Cells

Human breast carcinoma (MCF7 and MDA-MB-231) and renal carcinoma (Caki-1 and ACHN) cell lines were purchased from the American Type Cell Culture Collection (ATCC). CRBM-1990 cells were isolated and characterized in our laboratory from bone metastasis of renal cell carcinoma [41]. Breast and renal carcinoma cell lines were maintained in Iscove’s modified Dulbecco’s medium (IMDM) (Sigma-Aldrich, St. Louis, MO, USA) supplemented with 10% heat-inactivated fetal bovine serum (FBS) (Euroclone, Milan, Italy), plus 100 units/mL penicillin, and 0.1 mg/mL streptomycin (Life Technologies, Carlsbad, CA, USA) (complete IMDM). Cells were cultured at 37 °C in a humidified atmosphere of 5% CO_2_. Culture medium at specific pH was obtained by adjusting the concentration of sodium bicarbonate according to the Henderson–Hasselbalch equation, as previously described [42]. Unless otherwise indicated, cells were maintained at pH 7.4. To model acidic conditions, the pH of the medium was adjusted to 6.5. At the end-point of each experiment, the pH of the culture supernatants was measured to confirm the maintenance of the prefixed pH values during the incubation period by using a microelectrode (pH 301, HANNA Instruments, Woonsocket, RI, USA).

#### 2.1.2. Osteoclasts (OCs)

OC cultures were derived from fresh buffy coats of two healthy donors (AVIS, Bologna, Italy), as previously described [28,43]. Peripheral blood mononuclear cells (PBMC) were isolated on Ficoll–Histopaque gradient (GE Healthcare, Little Chalfont, Buckinghamshire, England), washed with Phosphate buffered saline (PBS), resuspended in Dulbecco’s Modified Eagle medium (DMEM) high glucose (Euroclone, Milan, Italy) supplemented with 10% heat-inactivated characterized FBS (Celbio), plus 100 units/mL penicillin, and 0.1 mg/mL streptomycin (complete high glucose DMEM), and plated in 8-well chamber slides (3 × 10^6^ cells/cm^2^). PBMC were incubated at 37 °C in a humidified atmosphere of 5% CO_2_ to obtain the monocyte adhesion. After 2 h, nonadherent cells were removed, and the medium was replaced with fresh complete high glucose DMEM medium added with 50 ng/mL RANKL and 10 ng/mL M-CSF (both from Peprotech, London, England) (pro-osteoclastogenic medium).

After 7 days of culture, cells were analyzed for tartrate-resistant acid phosphatase (TRACP) expression (Acid Phosphatase, Leukocyte kit, Sigma-Aldrich, St. Louis, MO, USA), according to the manufacturer’s protocols, dark-incubated with 2.25 μg/mL Hoechst 33258 (Sigma-Aldrich, St. Louis, MO, USA) at room temperature for 10 min, and visualized with a Nikon Eclipse E800M fluorescence microscope to verify OC differentiation. Only TRACP-positive cells with more than three nuclei were considered as OCs.

### 2.2. Extracellular Acidification Assay

We measured the pH of the culture medium using a microelectrode (pH 301, HANNA Instruments), as previously described [13]. Cells (16 × 10^6^) were washed twice in pHMed solution (80% normal saline, 10% unbuffered RPMI-1640 (Sigma-Aldrich), and 10% FBS), and incubated in pHMed in suspension for 3 h at 37 °C. Cells were then centrifuged (10 min at 500× *g*), and the supernatant was collected for extracellular pH (pHe) measurement. pHe was immediately quantified by a digital pH meter (pH 301, HANNA Instruments, Woonsocket, RI, USA). The difference in pH of pHMed solution alone (negative control), and the cell supernatants were analyzed and expressed as pH decrement. The experiment was repeated with six biological replicates.

### 2.3. Invasion Assay

Invasion assay was performed using transwells with 8 µm pores that were precoated with 1:3 diluted matrigel (BD Biosciences, Franklin Lakes, NJ, USA). Cells (1 × 10^5^) were diluted in 200 μL of IMDM containing 0.1% bovine serum albumin (BSA, Sigma-Aldrich) and seeded into transwells. In the lower compartment, 800 μL of complete RPMI-1640 at pH 7.4 or pH 6.5 were placed. Cells were then incubated in 5% CO_2_ at 37 °C and allowed to migrate for 72 h. At the end-point, invaded cells were fixed in methanol, stained with crystal violet solution, and counted from nine random fields (20× lens) in each well. The experiment was performed with three technical replicates.

### 2.4. Acridine Orange Uptake and Confocal Spectral Analysis of the Lysosomal pH

Differentiated OCs on the 7th day of culture or carcinoma cell lines at semi-confluence were dark-incubated with 1 μg/mL AO (Sigma-Aldrich) for 15 min at 37 °C. After one wash, x, y emission spectra from a confocal section within a living cell were recorded using a confocal laser microspectrofluorimeter (Nikon, TI, Minato, Tokyo, Japan), equipped with an argon-ion laser. Cells were focused with a 40× lens, 1.3 NA (S Fluor, Nikon, Minato, Tokyo, Japan), and excited at 457 nm. The resulting fluorescence emission in the 500–700 nm range was collected. For intracellular measurements of AO emission, the pinhole size was fixed to a diameter of 54 μm. To characterize the profile of AO emission spectra, the red band contribution (R%) within the whole emission spectrum was calculated as follows: R% = 100 × I655/(I655 + I530), where I655 and I530 are the green (520–540 nm) and the red (645–665 nm) integrated emission intensities, respectively [44]. The R% was calculated for all the acidic organelles within a single cell, and the average R% of acidic organelles of one single cell was considered. We averaged the measured signals from all the lysosomes in at least twelve single cells.

For the lysosome stability assay, we used real-time imaging of cells stained with AO and quantified the lysosome integrity after sensitizing them to photo-oxidation [45]. The photo-oxidation-induced loss of the lysosomal pH gradient and leakage of AO to the cytosol from individual lysosomes were quantified as a ‘loss of red dots’ or as a decrease in red and increase in green fluorescence. Briefly, sub-confluent MDA-MB-231 and differentiated OCs were incubated with 5 μg/mL AO for 15 min at 37 °C. Cells were then washed, exposed to blue light, and analyzed by time-lapse confocal imaging in IMDM supplemented with 3% FBS. Laser scanning micrographs were captured every 20 s for 600 s on a confocal laser microspectrofluorimeter (Nikon, TI) in two channels defined by bandpass filters for 531.24–539.25 nm (green) and 641.39–649.41 (red) light. Cells from at least three predefined areas were subsequently analyzed by the integrated Nis elements software to quantify the recorded green and red fluorescence intensities. Fluorescence intensities for each time-point were then normalized to the ratio to *t* = 0 s.

### 2.5. AO Uptake in 3D Tumor Spheroids

To obtain 3D tumor spheroids, an agarose mold was fabricated using a MicroTissues^®^ 3D Petri Dish^®^ micromold (Sigma-Aldrich, St. Louis, MO, USA) consisting of 81 circular cavities (9 × 9 arrays) with a diameter of 800 μm and depth of 800 μm. Molten agarose at 2% (500 μL) was cast on each of the micromolds and left for 20 min to set. Then the agarose molds were removed and placed in 12-well plates. The MDA-MB-231 breast carcinoma cell lines were then seeded in 81-well agarose molds at a density of 1.2 × 105 cells/mold in 190 µL of medium. To promote cell aggregation into spheroids, medium was supplemented with 10% FBS; cells were cultured at 37 °C in a humidified atmosphere of 5% CO_2_ for 7 days. Spheroids were then treated with 50 µg/mL AO (Sigma-Aldrich) for 30 min at 37 °C in complete medium. After one wash in PBS, fresh medium was added to the culture, and spheroids were then analyzed by confocal microscopy.

To evaluate the uptake of AO in the 3D tumor spheroids, we used laser scan confocal channel modality resonant, multiphoton, laser λ 800 nm, channel 2: laser power 0.7, PMT HV 88, PMT Offset −42 (emission wavelength: 525); channel 3: laser power 0.7, PMT HV 84, PMT Offset −51 (emission wavelength: 575). We used immersion objective 25×, line average 4, scan speed 7.5, and zoom 1.5.

### 2.6. In Vitro Cell Viability Assays

MDA-MB-231 cell viability after RDT with AO (AO-RDT) was determined using the Alamar blue test. Briefly, cells were seeded into 96-well plates (7.5 × 10^3^ cells/well) in complete IMDM. After 24 h, the medium was changed with complete medium containing 1 μg/mL AO, and cells were incubated for 15 min at 37 °C. Cells were then exposed to 1 and 5 Gy of X-ray. The irradiation conditions selected were a distance of 100 cm from the focus to the specimen, an irradiation rate of 1.40 Gy/min, a temperature of 21 °C, and an atmospheric pressure of 1005 hPa. At the end of RDT, the medium was discarded and replaced with a fresh complete medium. After 48 h of incubation, the cell culture medium was replaced with a fresh medium containing 10% Alamar Blue (Invitrogen, Waltham, MA, USA). The same solution was placed in an empty well and used to detect the background fluorescence (blank). The plates were incubated at 37 °C in a humidified atmosphere of 5% CO_2_ for 4 h. After cell incubation, the supernatant was transferred to a new plate and the fluorescence was measured with a microplate reader (Tecan Infinite F200pro) using an excitation wavelength of 540 nm and emission wavelength of 590 nm. Data were expressed as relative fluorescence units (RFU) after blank subtraction. The experiment was repeated twice with four technical replicates.

OC viability after AO-RDT treatment was evaluated by counting TRACP-positive multinucleated cells. Briefly, differentiated OCs were obtained by culturing mononuclear precursors isolated from fresh buffy coats of two healthy volunteers in the pro-osteoclastogenic medium for 7 days. After this period, OC differentiation was confirmed by analyzing cell TRACP expression and multinuclearity, as previously described. Mature OCs were exposed to the same protocol of treatments above described, and TRACP-positive multinucleated cells were counted to assess cell viability. At least two technical replicates were performed for each experiment.

### 2.7. In Vivo Study

#### 2.7.1. Omeprazole Systemic Administration

Procedures involving animals and their care were conducted with institutional approval and conformed to national and international laws and policies (EEC Council Directive 86/609, OJ L 358, 1, 12 December, 1987; Italian Legislative Decree 116/92, Gazzetta Ufficiale della Repubblica Italiana n. 40, 18 February 1992; NIH Guide for the Care and Use of Laboratory Animals, NIH Publication No. 85-23, 1985).

Four-week-old female Balb/cnu/nu mice were anesthetized with ketamine/xylazine (75 mg/kg body weight + 15 mg/kg body weight, respectively), injected monolaterally with MDA-MB-231 (5 × 10^4^ cells/10 µL PBS) in the medullar cavity of the left tibia and then treated intraperitoneally with vehicle (0.9% NaCl) or omeprazole (Sigma-Aldrich, 40 mg/kg body weight). Treatment was administered 5 days per week for 3 weeks, starting when osteolysis became apparent by X-ray analysis (curative protocol). To calculate the entity of osteolytic lesions at 21 days we anesthetized mice as described, laid them flat on an X-ray-sensitive film, and exposed them in an X-ray cabinet (Faxitron, Tucson, AZ, USA) to obtain standard 2D X-ray scans. After developing the films, we took high-resolution images of them and analyzed osteolytic lesions by manually selecting the osteolytic areas using the software ImageJ (NIH).

#### 2.7.2. Radiodynamic Therapy with Acridine Orange (AO-RDT)

The animal experiment was conducted by Pharmatest Services, with the approval of the National Committee for Animal Experiments (license number ESAVI-2077-04 10 07-2014). Five- to six-week-old female athymic nude mice (Hsd: Athymic nude, Foxn1nu; Envigo) were exposed to analgesic (Temgesic; buprenorphine, 0.1 mg/kg body weight) at least 30 min before the inoculation and then injected with MDA-MB-231 (1 × 10^5^ cells in 20 µL of PBS) into the bone marrow of right proximal tibia. Two weeks after the inoculation, the tumor-induced bone changes were quantified by X-ray imaging, and the lesion areas were used to stratify the animals into treatment groups with similar mean total osteolytic bone lesion areas. Briefly, animals were imaged in a prone position with the Faxitron Specimen Radiographic System MX-20 D12 (Faxitron). One radiograph showing both hind limbs per animal was taken on each X-ray occasion (34 kV, 7 s, magnification 2×). After animal stratification, a single dose of AO (Sigma-Aldrich, 4 mL/kg, resulting in a dose of 5 mg/kg) or vehicle (4 mL/kg PBS) was administered intraperitoneally, and 2 h after the dosing, 5 Gy radiation of X-ray was administered to the mice. For irradiation, the mice were anesthetized with isoflurane (Attane Vet 1000 mg/g Isoflurane, Piramal Healthcare). The irradiation was directed to the tumor-bearing tibia by applying a metallic cover on the mice with a round hole for radiations beams to access the area of the tumor (diameter for the hole in the cover was 10 mm, resulting in a maximal area to be irradiated to be 78.5 mm^2^). The clinical condition of the mice was followed daily by cage-side observations, and animal weight was recorded twice a week. At 4 weeks after intratibial inoculation, the development of bone lesions was quantified again by radiography imaging, the mice were sacrificed with CO_2_ and the death was confirmed by cervical dislocation. The tumor-bearing tibias were collected to 10% neutral buffered formalin (NFB) for histological evaluation of tumor area and the quantification of OCs.

#### 2.7.3. Histological Analysis

Paraffin-embedded tumor-bearing tibias were prepared for histology. The tumor-bearing tibias were de-calcified in ethylenediaminetetraacetic acid (EDTA) for two weeks. After decalcification, the samples were dehydrated in an ascending series of ethanol concentrations, clarified in xylene, and embedded in paraffin (TEK III Paraffin Wax, Sakura). Then midsagittal 4 µm sections were obtained from each animal. For subsequent histological stainings, the sections were deparaffinized in xylene and rehydrated in a series of descending ethanol concentrations.

Sections were stained for hematoxylin, eosin, and Orange G (H&E-Orange G; Leica Biosystem and Sigma-Aldrich) for evaluation of the tumor area and for TRACP (Sigma-Aldrich) to quantify OC number.

#### 2.7.4. Histomorphometric Analysis

The H&E-Orange G and TRACP stained sections were obtained from each tumor-bearing tibia, scanned with a digital slide scanner, and analyzed with Pannoramic Viewer (3D Histech). Tumor area was defined in each section from the growth plate to 5 mm distance to the proximal tibia. These areas were defined for each section and analyzed by color-thresholding. OC number and tumor–bone interface (TBI) were defined in each TRACP stained section and analyzed as the total number of OCs and the number of OCs on TBI.

### 2.8. Statistics

Because of the small number of observations, we did not consider the data normally distributed and therefore used nonparametric tests. For in vitro experiments, we performed statistical analyses using GraphPad Prism 7.0 software (GraphPad Software, San Diego, CA, USA). For differences between the two groups, we used the Mann–Whitney U test. For in vivo studies, we performed statistical analyses by statistical software R (version 3.3.2 or newer, 31 October 2016, www.r-project.org). The end-point parameters were analyzed using fixed-effects models. The parameters with multiple measurements per subject (body weight and radiographic analyses) were analyzed using mixed or fixed-effects models. If necessary, we transformed data using an appropriate transformation (e.g., logarithmic or square root transform) before the analysis to obtain the proper fit of the model to the data. We adjusted the obtained *p*-values for multiple comparisons. For analyzing the interaction between AO and irradiation, we used the analysis of variance.

For all the experiments, we expressed values as the mean ± standard error of the mean (SEM) and considered only *p* < 0.05 values statistically significant.

## 3. Results and Discussion

### 3.1. Tumor Acidosis in BM Enhances Local Carcinoma Aggressiveness

Highly proliferative cancer cells are strongly glycolytic and thus secrete substantial amounts of protons/lactate into the extracellular environment (Warburg effect) [46]. The resulting extracellular acidosis is a hallmark of several solid tumors, including BM from advanced carcinomas [4,5].

Tumor cells adapt to and take advantage of extracellular acidosis to enhance their aggressiveness [47,48,49], as acidosis increases the secretion and activity of enzymes that degrade the extracellular matrix (ECM) [47]. Among them, cathepsin B and MMP, especially MMP-2 and MMP-9, are strongly associated with increased tumor growth and invasion in vivo [50,51]. In bone, this mechanism may be even more crucial for cancer invasiveness and tumor expansion due to its mineralized ECM matrix. Furthermore, a low pH exacerbates tumor-induced osteolysis by directly stimulating OC-mediated bone resorption [10,11,12], or by enhancing an inflammatory pro-osteolytic phenotype in osteogenic cells [13].

**Figure 1:**
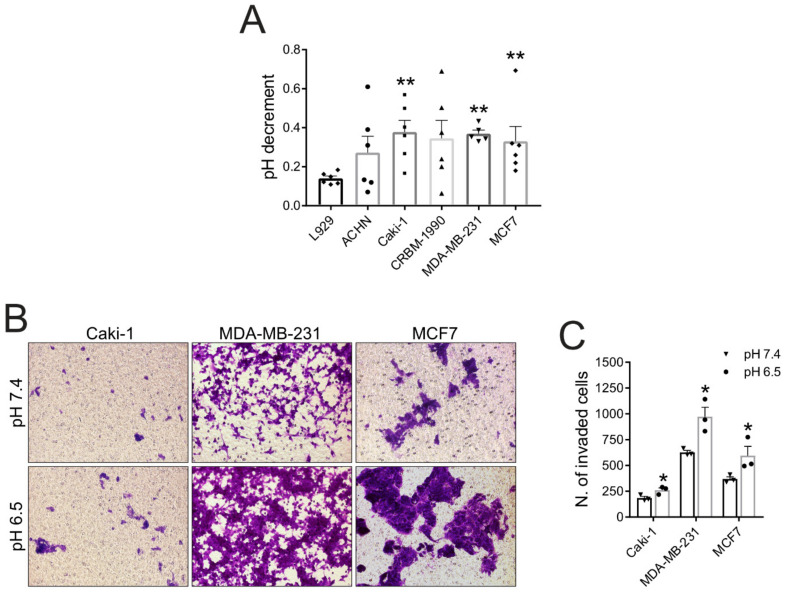
**Tumor acidosis in BM enhances local carcinoma aggressiveness**. (**A**) Carcinoma cells acidify the extracellular environment as assessed by the quantification of pH decrement of cell culture supernatant (mean ± SEM, *n* = 6, ** *p* < 0.01); (**B**) Representative images of carcinoma cell invasion assay using matrigel-coated transwells. Cells migrated towards acidic (pH 6.5) or neutral (pH 7.4) medium were stained with crystal violet (20× lens); (**C**) Quantification of migrated cells in the assay shown in (**B**) (mean ± SEM, *n* = 3, * *p* < 0.05 vs. pH 7.4).

Hence, to validate tumor acidosis as a possible target in BM, we first verified the acidification activity of carcinoma cells that usually metastasize to the bone. For this aim, we used carcinoma cell lines with a high tendency to metastasize to the bone. These have been isolated from both renal (ACHN, Caki-1, CRBM-1990) and breast cancer (MDA-MB-231 and MCF7). We had previously characterized all the cell lines used for their ability to induce OC differentiation and activity [13]. As expected, all the examined carcinoma cells were able to strongly acidify the extracellular space, as revealed by the quantification of proton concentration in the unbuffered cell culture supernatant (Figure 1A). In particular, Caki-1, MDA-MB-231, and MCF-7 showed a significantly higher acidification ability than the normal L929 fibroblasts (*p* = 0.0043). We then validated the role of acidosis in directly prompting the invasive potential of carcinoma cells: the presence of a low pH (pH 6.5) in the lower compartment of a matrigel-coated transwell strongly induced both breast and renal carcinoma cells to cross the matrigel-membrane layer, thus enhancing their invasive potential (Figure 1B,C).

### 3.2. In Vivo Targeting of Intratumoral Acidification by V-ATPase Blockage Does Not Reduce Osteolysis

We have previously shown that breast and renal carcinoma cells acidify the extracellular space through the expression of V_1_B_2_, V_0_C, and V_1_G_1_ subunits of the V-ATPase [13]. By pumping the excess of protons outside the cytosol, proton pumps, such as V-ATPases, are crucial players in progression to a metastatic and invasive cell phenotype [52,53,54] and represent a suitable therapeutic target [55]. As evidence, V-ATPase inhibitors have been recently and extensively considered as anticancer drugs in different cancer cells, including carcinoma cells [42,56,57,58,59,60,61,62,63].

V-ATPase is not exclusively expressed by tumor cells since it is ubiquity detected on the membrane of endolysosomal organelles and the plasma membrane of normal specialized cells, such as OCs, that strongly acidify the extracellular space to resorb bone [62,63,64,65,66]. The selective inhibition of V-ATPase prevents in vivo bone loss [67]. Thus, in the specific case of BM, the impairing of V-ATPase in both carcinoma cells and OCs has the potential to advantageously prevent tumor progression and tumor-induced OC activity at the same time. According to this hypothesis, as a preliminary experiment, we administered omeprazole, a gastric H^+^/K(^+^)-ATPase inhibitor that can also block V-ATPase [42], to a xenograft model of BM from breast carcinoma. However, in contrast with previous in vitro data [66,68], although we observed a nonsignificant trend of reduction at 21 days, this type of approach was insufficient to reduce osteolysis in vivo (Figure S1A,B). Notably, the use of proton pump inhibitors (PPIs) to treat osteolysis is highly controversial. Long-term administration in animal models and patients may interfere with intestinal calcium absorption, ultimately resulting in bone fractures, also affecting absorption of vitamin B12, iron, and magnesium, thus causing deficiency of these essential elements [69,70,71,72]. On the contrary, in other in vivo and clinical studies, omeprazole has been shown to reduce bone resorption and slow down the progression of osteoporosis [67,73,74].

We then concluded and considered that: (1) in our model, omeprazole is not effective in inhibiting bone resorption; (2) omeprazole administration may induce long-term side effects; (3) extracellular acidosis may derive from a highly redundant system of different ion/proton transporters that cannot be effectively blocked through the impairment of a single target [5]. We thus moved from the selective targeting of one single proton pump/transporter to a different antiacid therapeutic strategy. We therefore considered AO, a well-known dye for lysosome staining in live cells, due to its tropism towards the acidic compartments, and for its use in the lysosome stability assay, due to its ability to unveil lysosomal fragility through the induction of LMP.

### 3.3. Breast Carcinoma Cells Are More Sensitive to Lysosomal Death than OCs

Several reports have demonstrated that lysosomes of cancer cells are more fragile and prone to LMP than their normal counterpart, making them good therapeutic targets [22,75,76,77]. We then wondered if targeting lysosomes could be a useful antiacid therapeutic strategy for treating BM. We first evaluated the level of AO uptake into lysosomes of both carcinoma cells and OCs.

**Figure 2:**
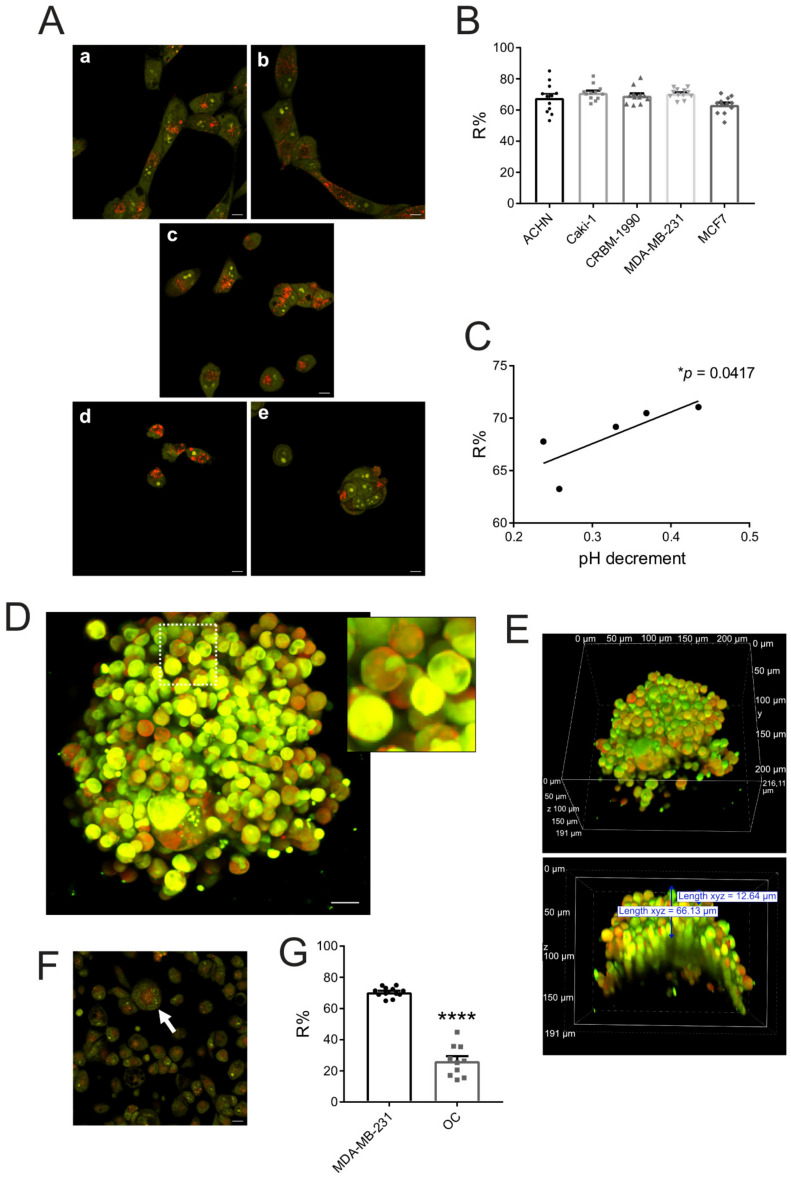
**Carcinoma cells show greater lysosomal uptake of AO than osteoclasts.** (**A**) Representative confocal images of AO accumulation into lysosomes of ACHN (**a**), Caki-1 (**b**), and CRBM-1990 (**c**) renal carcinoma cells, and MDA-MB-231 (**d**) and MCF7 (**e**) breast carcinoma cells; red signals correspond to the cell compartments with the highest concentration of AO. Scale bar 20 µm; (**B**) Quantification of the red band contribution (R%) within the whole emission spectrum of AO by spectral confocal microscopy in the lysosomes of cells shown in (**A**) (mean ± SEM, *n* = 12); (**C**) Correlation of R% with the extracellular acidification activity of carcinoma cells (average of pH decrement as assessed by pHmed assay) (mean ± SEM, * *p* = 0.0417; (**D**) Merge confocal z-stack images of AO internalization of a representative live tumor spheroid of MDA-MB-231 breast carcinoma cells (191 stacks, 1 µm); red signals correspond to lysosomes (scale bar: 20 µm). An enlargement of an area of the image is visible in the frame; (**E**) Volume render of the same MDA-MB-231 spheroid shown in (**D**). In the lower panel, a cross-section of the tumor spheroids was shown to highlight that AO was uptaken up to 5–6 cells deep from the edge of the spheroid (red signal detected at 66.13 µm from the spheroid border, cell diameter 12.64 µm); (**F**) Representative confocal images of AO accumulation into lysosomes of an osteoclast (multinucleated cell indicated by white arrow); scale bar: 20 µm; (**G**) Comparison of R% of MDA-MB-231 and osteoclasts (OCs) (mean ± SEM, *n* = 12 for MDA-MB-231 and *n* = 10 for OCs, **** *p* < 0.0001).

When highly concentrated, like in very acidic lysosomes, AO takes the form of di- and oligomeric aggregates that, once excited, emit a red signal; when less concentrated, AO takes the monomeric form that emits a green signal. After AO excitation, we then measured the red band contribution in the small dots, corresponding to acid intracellular organelles, with respect to the green band signal (R%) in the same dots (Figure 2). Notably, we observed AO accumulation in intracellular organelles in all the different carcinoma cell lines (Figure 2A,B). As a negative control for AO-specific localization in the acidic intracellular compartment, we used cells fixed with methanol and then incubated with culture medium. Indeed, alcohol fixation causes cell membrane permeabilization. As a consequence, intracellular pH (cytosolic and organelle) converts to a pH value equal to that of the culture medium (pH 7.4) (Figure S2). As expected, AO-treated fixed cells did not internalize highly concentrated AO. In these cells, AO mainly bound nuclear DNA and nucleolar RNA as a monomer (green fluorescence, 525 nm) (Figure S2, upper panels). Conversely, in live cells, AO accumulated into acidic intracellular organelles as red fluorescent granules (Figure S2, lower panels). We also confirmed the lysosome localization of polymeric AO by analyzing and quantifying the localization with a specific stain of lysosomes (Lysotracker, Figure S3).

Furthermore, the AO concentration in lysosomes of carcinoma cells was pH dependent, as we observed a significant correlation between the R% and the extracellular low pH, as measured by an electrode (Figure 2C, *p* = 0.0417).

To study the in vitro uptake of AO in a more realistic setting, we also developed tumor spheroids from MDA-MB-231 breast carcinoma cells. Confocal analysis showed AO internalization and lysosomal intracellular accumulation (red signal) also in the 3D developed model (Figure 2D). The AO signal was detected up to 5–6 cells deep from the edge of the spheroid (Figure 2E).

Similar to carcinoma cells, OCs also accumulated AO into lysosomes (Figure 2F). However, AO accumulation was significantly lower than in cancer cells (Figure 2G, *p* < 0.0001), suggesting that OCs have less acidic lysosomes than carcinoma cells, or uptake lower concentrations of AO into the lysosomes from the extracellular space. This hypothesis is in agreement with previous evidence in vivo, both in animals and in human patients, showing a higher tropism of AO for malignant tissues compared to normal tissues [78,79,80,81].

**Figure 3:**
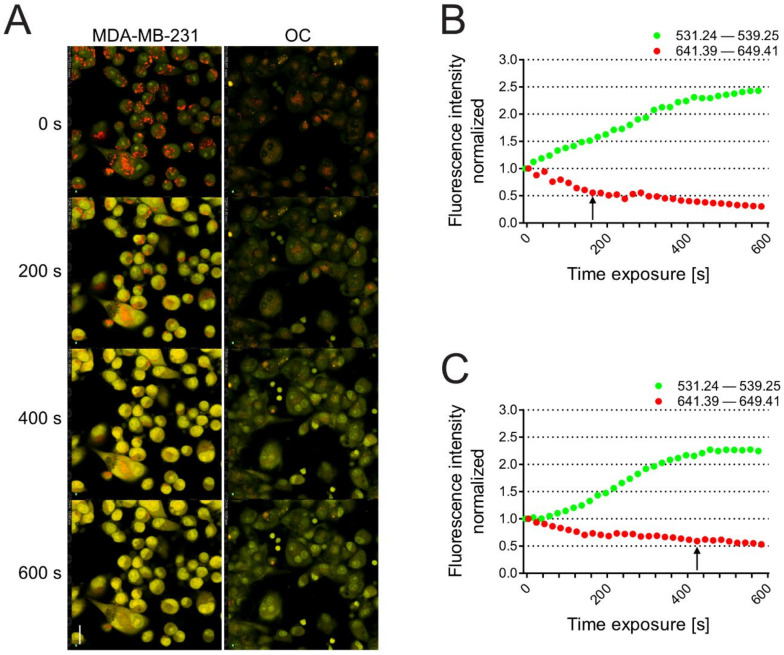
**AO lysosomal stability assay.** MDA-MB-231 and osteoclast (OC) cells were stained with AO for 15 min and then exposed to blue light from a confocal laser microspectrofluorimeter; laser scanning micrographs were captured every 20 s by using two channels, defined by bandpass filters for 531.24–539.25 nm (**green**) and 641.39–649.41 (**red**) light. (**A**) Representative images acquired by confocal microscopy at different time-points; scale bar: 20 µm. Quantification of red and green fluorescence in MDA-MB-231 (**B**) and OCs (**C**). At least two cells in the predefined areas from 31 independent frames were subsequently analyzed by the integrated Nis elements software (Nikon). Green and red fluorescence intensities for each time-point were normalized to the ratio of *t* = 0 s.

We then performed a lysosome stability assay in the same cells to measure LMP. In this case, once inside the lysosomes, the metachromatic AO sensitizes the lysosomal membrane to photo-oxidation by blue light [18]. Upon light-induced loss of the lysosomal pH gradient and subsequent leakage of AO into the cytosol, the emission spectra of AO shift from red to green over time [16], as it moves from acidic lysosomes to alkaline cellular compartments, such as the cytosol and nucleus [45,82,83]. Hence, loss of lysosomal integrity can be measured by quantifying the red and the green signal in the entire area of the cell as a ‘loss of red dots’ or as a quantitative rise in total green fluorescence. This assay has been already used in several studies, for example, to measure the lysosomal cell death induced by inhibitors of acid sphingomyelinase [84]. Carcinoma cells had weaker lysosome stability than OCs (Figure 3A and Videos S1 and S2 for carcinoma cells and OCs, respectively), as demonstrated by a greater increase in green (cytosolic) fluorescence and a quick decrease in red (lysosomal) dots (black arrow, Figure 3B). In contrast, OCs were more resistant to the LMP induced by excited AO, as they retained AO into lysosomes for a longer period (Figure 3C, black arrow).

### 3.4. AO-RDT Impairs Tumor-Induced Osteolysis and Cancer Cell Survival

The ability of AO to induce lysosomal leakage and cell death after excitation with blue light (AO-PDT) has been successfully combined with surgery for tumor excision as, in this case, the tumor site is easily exposed to light in the operating field [28,32,33,34,35,36]. However, BM typically results from the systemic spread of cancer cells and is rarely surgically treated. Therefore, we considered using radioactivated AO to treat BM. In addition to blue light, AO can be activated by X-rays (AO-RDT). AO-RDT exerts similar cytotoxic effects while having greater tissue penetration than AO-PDT. Hence, AO-RDT may be more suitable for the noninvasive treatment of large, deep-seated tumors that grow within inaccessible sites [37,85], such as carcinomas metastasized to the bone, especially pelvis and spine that are inoperable regions. Furthermore, RDT may simultaneously and synergistically act as radiotherapy and PDT activator, maximizing the treatment effect [86,87]. An additional benefit of AO-RDT is that normal cells are radio-resistant rather than cancer cells. Besides AO-PDT, AO can also increase the production of toxic reactive radicals when exposed to low doses of X-ray radiation [86]. Of note, the dose of radiation applied to the tumor bed to excite AO is much lower than that adopted for standard radiotherapy, thus reducing or avoiding the associated acute and late side effects [30,31,39].

**Figure 4:**
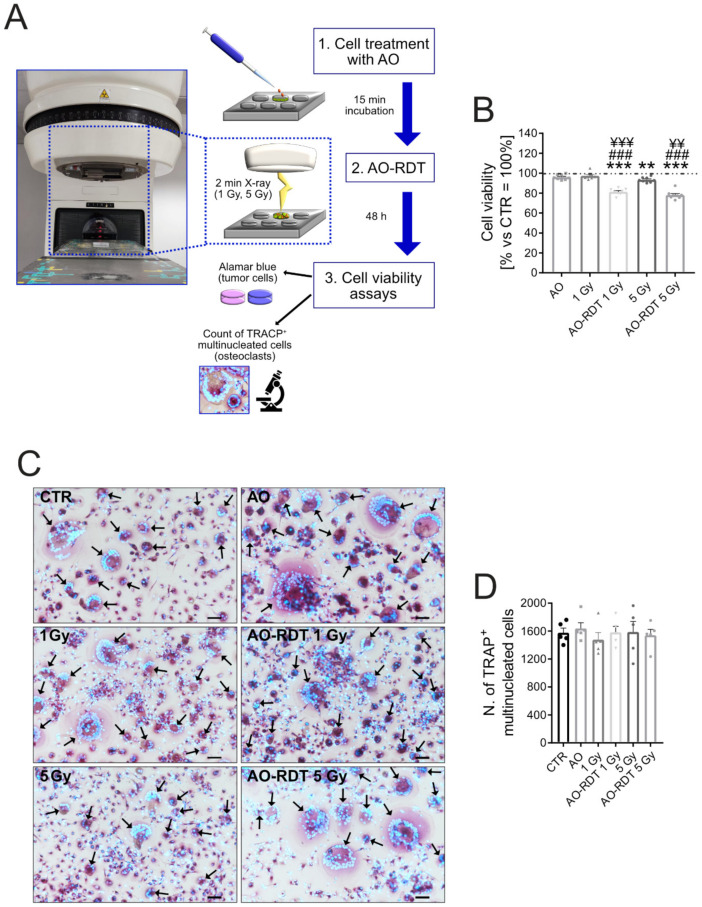
**In vitro radiodynamic therapy with AO.** (**A**) Representative image of the linear accelerator used for in vitro radiodynamic treatment with AO (AO-RDT); (**B**) Alamar blue assay to detect MDA-MB-231 cell viability 48 h after exposure to AO, 1 or 5 Gray irradiation, or AO combined with 1 or 5 Gray irradiation (AO-RDT 1 Gy and AO-RDT 5 Gy, respectively); not treated cells were used as negative control (CTR) (mean ± SEM, *n* = 8, ** *p* < 0.01 and *** *p* < 0.001 vs. CTR, ^###^ *p* < 0.001 vs. AO, and ^¥¥^ *p* < 0.01 and ^¥¥¥^ *p* < 0.001 vs. irradiation); (**C**) Representative images of the n. of TRACP^+^ multinucleated OCs 48 h after exposure to the different treatment conditions; scale bar: 25 μm. Nuclei were counterstained with Hoechst 33258; black arrows indicate TRACP^+^ multinucleated OCs; (**D**) Graph of the n. of TRACP^+^ multinucleated OCs 48 h after exposure to the different treatment conditions (mean ± SEM, *n* = 5).

We first investigated the in vitro effect of combination therapy on MDA-MB-231 breast carcinoma cells and OCs. We used a linear accelerator (Figure 4A) to radioactivate AO by low (1 Gy), or moderate (5 Gy) irradiation. As already demonstrated in an in vitro model of osteosarcoma [37], AO-RDT treatment, at both 1 Gy and 5 Gy doses, significantly reduced MDA-MB-231 viability compared with untreated cells (*p* = 0.0008), AO monotherapy (*p* = 0.0008), and radiation alone (*p* = 0.0008 for AO-RDT 1 Gy and *p* = 0.0011 for AO-RDT 5 Gy, Figure 4B). Interestingly, AO monotherapy had no effects on cell viability, whereas the higher dose of radiation (5 Gy) was significantly toxic as compared to untreated cells (Figure 4B, *p* = 0.0063). The same radiation dose was nontoxic for OCs (Figure 4C,D). These data confirmed that proliferating cancer cells are more sensitive to radiation than normal cells [88] also in our cell models. Furthermore, AO-RDT did not alter the number of mature OCs (Figure 4D), suggesting a selective cytotoxic activity towards carcinoma cells.

**Figure 5:**
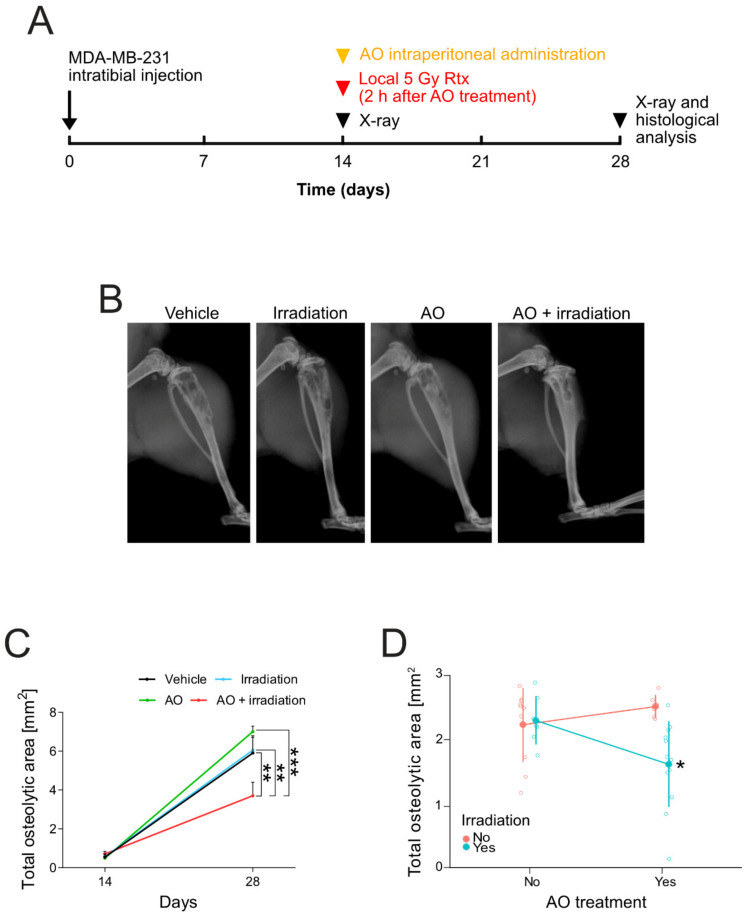
**In vivo radiodynamic therapy with AO in the xenograft mouse model of BM.** (**A**) Schematic representation of the in vivo study. On Day 0, mice underwent an intratibial injection of MDA-MB-231 cells. On Day 14, mice were imaged with X-ray and then treated with a single dose of AO or PBS (vehicle) by intraperitoneal administration, and 2 h after treatment, were exposed or not exposed to 5 Gy local irradiation. On Day 28, mice were imaged with X-ray, and tumor-bearing-tibias were collected for histological analyses; (**B**) Representative X-ray scans of osteolysis in the different experimental groups; (**C**) Total osteolytic lesion area by the quantification of X-ray imaging at different time-points (mean ± SEM, *n* = 10 for vehicle, *n* = 7 for irradiation, *n* = 6 for AO, and *n* = 12 for AO + irradiation groups, * *p* < 0.05, ** *p* < 0.01, and *** *p* < 0.001); (**D**) Interaction of AO and irradiation treatments on total osteolytic lesion area as evaluated by 2 × 2 factor analysis (mean ± SEM, *n* = 10 for vehicle, *n* = 7 for irradiation, *n* = 6 for AO, and *n* = 12 for AO + irradiation groups, * *p* < 0.05).

Most importantly, we obtained similar results also in the xenograft model of BM from breast carcinoma that we previously used to test omeprazole treatment. Two weeks after inoculation of MDA-MB-231 breast cancer cells, we administered AO to mice and exposed them to 5 Gy local radiation (Figure 5A). Both AO and AO-RDT treatments did not change the body weight (Figure S1) or cause evident organ toxicity in mice, suggesting that it was well tolerated and confirming its safety, as already demonstrated in long-term exposed mice and in human clinical trials to treat osteosarcoma and rhabdomyosarcoma [17,39,89]. At the end of the protocol, the combination treatment with AO and irradiation significantly decreased the total bone lesion area compared to vehicle (*p* = 0.0033), irradiation (*p* = 0.0040), and AO-treated mice (*p* < 0.001), as shown by the representative X-ray images (Figure 5B) and relative quantification graph (Figure 5C). Furthermore, by the analysis of variance, we found a significant interaction between the treatment with AO and irradiation when in combination (Figure 5D and Table 1).

**Figure 6:**
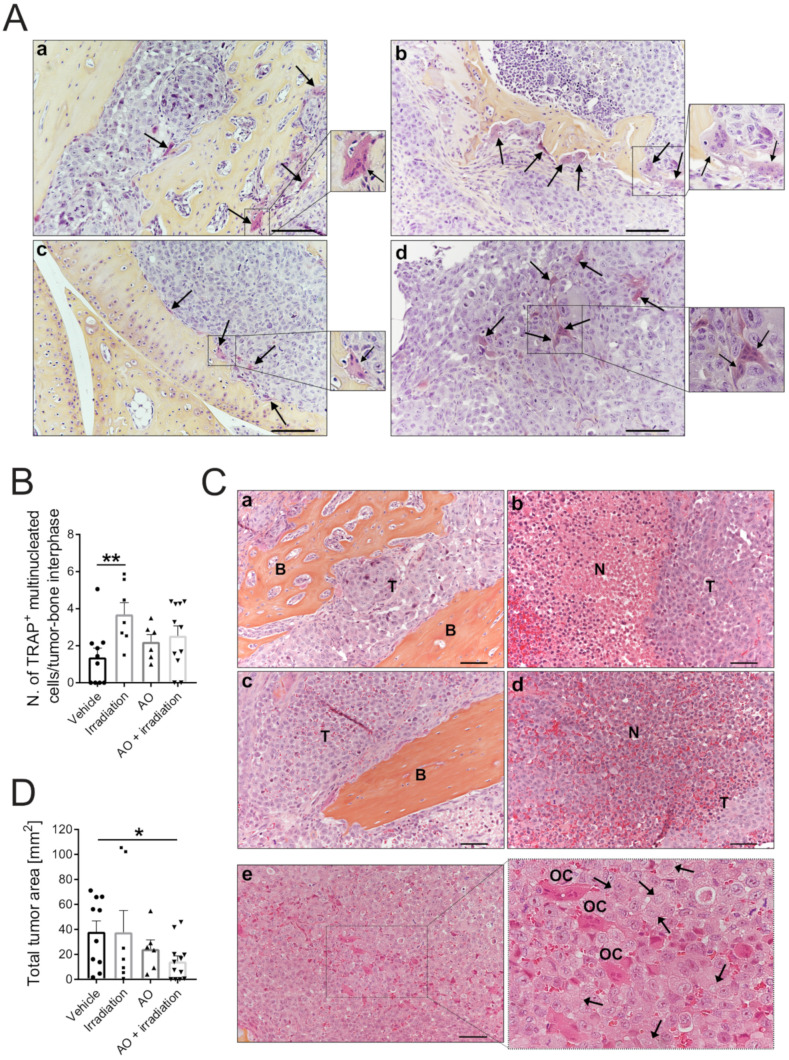
**Histological analysis of tumor-bearing-tibiae of mouse xenografts.** (**A**) Representative images of TRACP staining in paraffin tissue sections from mice treated with vehicle (PBS) (**a**), irradiation (vehicle plus 5 Gy irradiation) (**b**), AO (AO solution, 5 mL/kg) (**c**), or AO with irradiation (5 mL/kg AO plus 5 Gy irradiation) (**d**). TRACP^+^ multinucleated OCs are indicated by black arrows; scale bar: 100 µm; (**B**) Quantification of TRACP^+^ multinucleated OCs per tumor–bone interface for each group in the tissue sections shown in (**A**) (mean ± SEM, *n* = 10 for vehicle, *n* = 7 for irradiation, *n* = 6 for AO, *n* = 11 for AO + irradiation, ** *p* < 0.01); (**C**) Representative images of H&E-OrangeG staining for each group: vehicle (**a**), irradiation (**b**), AO (**c**), and AO with irradiation (**d**,**e**). H&E-OrangeG staining shows tumor (T) infiltration in bone (**B**), with extensive disruption and necrosis (N) of its parenchyma in the irradiation (**b**) and AO + irradiation (**d**) groups. The tissue section of an additional sample of tumor-bearing-tibia from mice treated with AO + irradiation (**e**) shows live osteoclasts (OCs) with regular nuclei and, on the contrary, deranged tumor tissue including several degenerating tumor cells with possible karyolysis (black arrows); (**D**) Total tumor area as quantified from the H&E-OrangeG stained histological sections from the predefined analysis area by color-thresholding (mean ± SEM, *n* = 10 for vehicle, *n* = 7 for irradiation, *n* = 6 for AO, *n* = 12 for AO + irradiation, * *p* < 0.05).

We then performed a histomorphometric analysis of the tumor-bearing tibiae of mice sacrificed at 28 days. AO-RDT did not alter the number of OCs at the bone–tumor interface, as demonstrated by TRACP staining on tissue sections (Figure 6A) and the relative quantification of TRACP-positive multinucleated OCs (Figure 6B). We thus excluded that the inhibition of osteolysis by AO-RDT was due to a direct cytotoxic activity of the treatment on OCs. Instead, we speculated that AO-RDT selectively targeted cancer cells, ultimately blocking their paracrine secretion that may induce OC bone resorption rather than OC differentiation. These results are in agreement with our previous in vitro data showing that AO-RDT is cytotoxic on cancer cells, and that carcinoma cells can accumulate AO at a higher concentration than OCs (Figure 2E) and are more sensitive to LMP (Figure 3) than OCs, suggesting that cancer cells are more prone to AO-RDT-induced lysosome-dependent cell death.

In line with these findings, H&E-orange staining on tumor sections from mice exposed to AO-RDT showed extensive necrosis in the tumor parenchyma. In particular, we observed deranged tumor tissue characterized by several degenerating cancer cells with loss of nuclear staining, suggesting karyolysis (Figure 6C, black arrows). In contrast, OCs did not appear to be affected by AO-RDT and showed nuclei without morphological evidence of cell death (Figure 6C). Some necrosis was occasionally evident also in the irradiated tissues (Figure 6C). As a further confirmation of the tumor-selective toxicity of AO, in xenografts, AO-RDT significantly decreased the total bone–tumor area compared to vehicle (Figure 6D, *p* = 0.0415). In contrast, we did not detect significant differences in response to AO monotherapy.

## 4. Conclusions

Tumor acidification is an important mechanism that drives cancer aggressiveness in BM since it increases the invasiveness of tumor cells and the resorption activity of OC. However, targeting the most crucial proton pump, the V-ATPase, was not effective in vivo. In contrast, the use of radioactivated AO seems to offer a novel and effective therapeutic option. AO is widely known to accumulate within acidic lysosomes of tumor cells and to induce lysosomal membrane-dependent cell death in cells that are particularly susceptible to this event, such as tumor cells. According to our data, AO-RDT is selectively toxic in carcinoma cells and significantly inhibits tumor-induced osteolysis, both in vitro and in vivo, even though it does not affect OC viability. Of note, according to the current available data, both local and systemic AO does not induce toxicity or carcinogenesis [17]. Therefore, this innovative therapeutic approach appears to be very promising for the treatment of BM together with standard antiresorptive agents.

## Figures and Tables

**Table 1:** **Total lesion area, relative (mm^2^). Analysis of variance.** Prior to statistical analysis, the data were transformed using square root transform. NA, not applicable.

Effect	Sum of Squares	Degrees of Freedom	*F*-Value	*p*	Interpretation
Acridine orange	0.35	1	1.09	0.3037	
Irradiation	0.02	1	0.07	0.7975	
Interaction	2.11	1	6.69	0.0146	Interaction
Residuals	9.79	31	NA	NA	

## Data Availability

Data is contained within the article and Supplementary Material.

## Supplementary Materials

The following supporting information can be downloaded at: https://www.mdpi.com/article/10.3390/biomedicines10081904/s1, Figure S1: Specific targeting of V-ATPase is not effective to impair BM-induced osteolysis; Figure S2: Internalization of AO into fixed and live cells; Figure S3: Colocalization of AO polymer and lysotracker in live cells; Figure S4: Mouse body weight development; Video S1: Lysosome stability assay on MDA-MB-231; Video S2: Lysosome stability assay on osteoclasts.

## Abbreviations

AO: acridine orange; AO-RDT: radiodynamic therapy with AO; brain-derived neurotrophic factor (BDNF); BM: bone metastases; DMEM: Dulbecco’s modified Eagle medium; ECM: extracellular matrix; EDTA: ethylenediaminetetraacetic acid; FBS: fetal bovine serum; IL-6: interleukin 6; IL-8: interleukin 8; IMDM: Iscove’s modified Dulbecco’s medium; LMP: lysosome membrane permeabilization; M-CSF: macrophage colony-stimulating factor; MMPs: matrix metalloproteinase; NFB: neutral buffered formalin; OC: osteoclasts; PBMC: peripheral blood mononuclear cell; PBS: phosphate buffered saline; PDT: photodynamic therapy; RANKL: receptor activator of nuclear factor-kappa b ligand; RDT: radiodynamic therapy; RFU: relative fluorescence units; SEM: standard error of the mean; TBI: tumor-bone interface; TNF: tumor necrosis factor; TRACP: tartrate-resistant acid phosphatase; V-ATPase: vacuolar (H+)-ATPase.
