# Supplementary material for: Radiodynamic Therapy with Acridine Orange Is an Effective Treatment for Bone Metastases
Source: Biomedicines. 2022 Aug 5;10(8):1904. doi: 10.3390/biomedicines10081904 (PMC9405350; doi:10.3390/biomedicines10081904)
Supplement: Supplementary file 1 [file biomedicines-10-01904-s001.zip › Supplementary Materials.pdf]

Supplementary Materials and Methods

Confocal analysis

For the comparison of AO uptake/localization in live and fixed cells, we used laser scan confocal, channel modality Galvano, 487.2 λ laser power 1.6, PMT HV 29, PMT Offset -12 (emission wavelength: 525); 561.3 λ laser power at 2.1 (emission wavelength: 595), PMT HV 144, PMT Offset -3; TD PMT HV 91 and PMT offset 0. We used objective 40x, line average 8, pinhole size 22.99 µm, scan speed 7.5, and zoom 2.0.

For co-localization analysis between AO and LysoTracker™ Green signals, living adherent cells at semi-confluence were incubated for 30 minutes at 37°C and 5% CO_2 in complete medium added with LysoTracker™ Green DND-26 (500 nM, Molecular Probes, Life Technologies). At the end of the incubation period, cells were washed with PBS and then dark-incubated with 1 µg/mL AO (Sigma-Aldrich) for 15 minutes in complete medium at 37°C and 5% CO_2. Cells were then analyzed using a confocal microscope (A1R, Nikon) with laser scan confocal, channel modality Galvano, laser λ 487.2 nm, laser power 1.2, emission wavelength (virtual filter): 493.2-511.2 (channel 1) and 631.2-655.2 (channel 2), PMT HV 159, gain 15 for channel 1 and 8 for channel 2, TD: PMT HV 81 e PMT Offset -58; we used immersion objective 25x, line average 4, pinhole size 25.54 µm, scan speed 0.063, and zoom 2.392.

Supplementary Figures

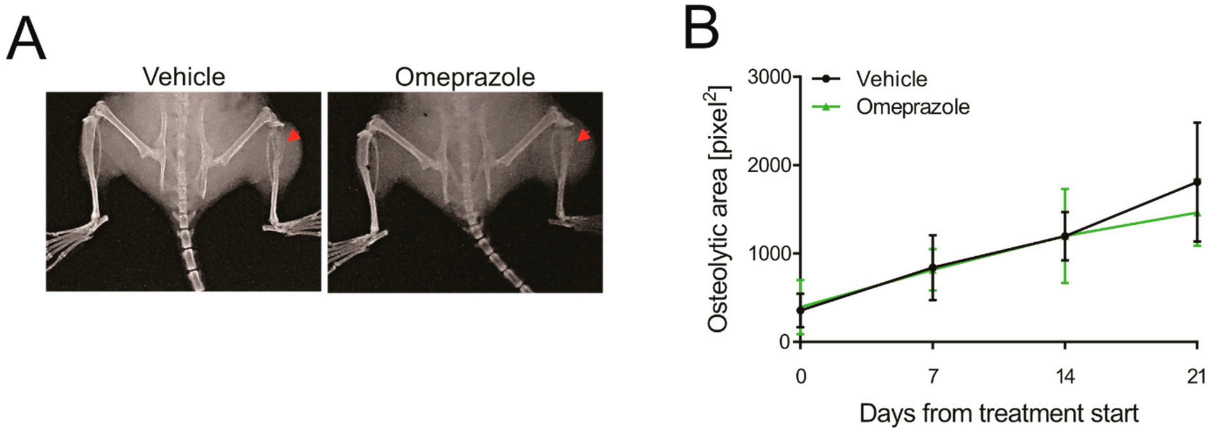

Figure S1. Specific targeting of V-ATPase is not effective to impair BM-induced osteolysis. (**A**) Representative X-ray scans in mouse xenografts after 21 days of treatment with omeprazole (40 mg/kg) or vehicle (red arrows correspond to osteolytic area); (**B**) Quantification of the osteolytic areas over time in mice treated with omeprazole or vehicle (mean ± SEM, *n*=6 vehicle, *n*=7 omeprazole).

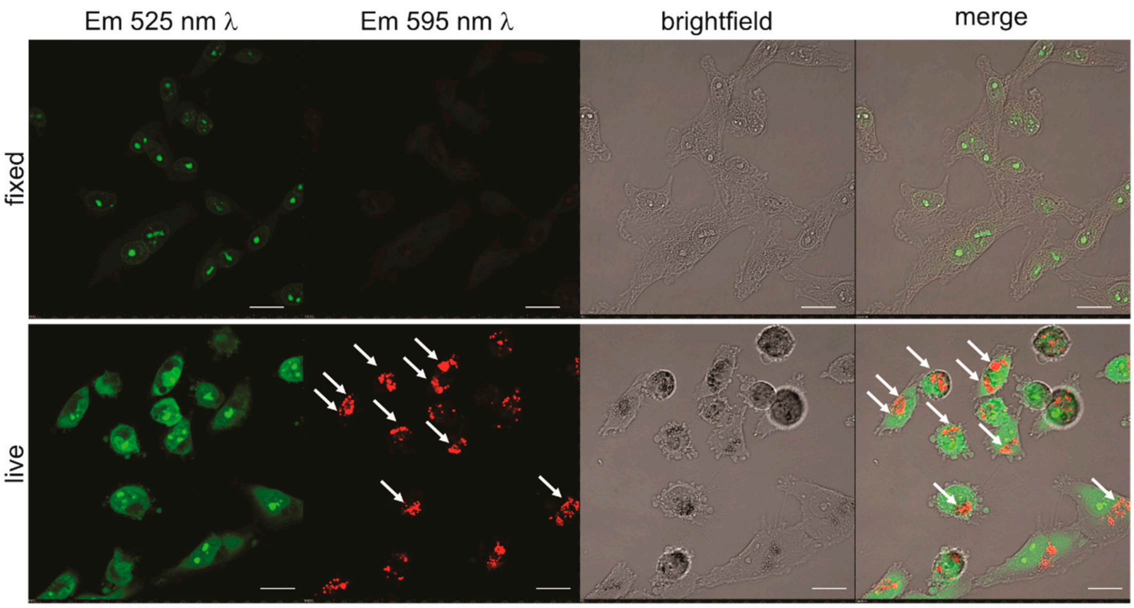

Figure S2. Internalization of AO into fixed and live cells. Merge of confocal and transmitted light images of fixed (upper panel) and live (lower panel) MDA-MB-231 that were pretreated with AO (scale bar 20 μm). White arrows indicate lysosomal accumulation of AO into live cells. We used fixed cells as negative control for AO internalization to exclude any possible contribution coming from the *in vitro* conditions used.

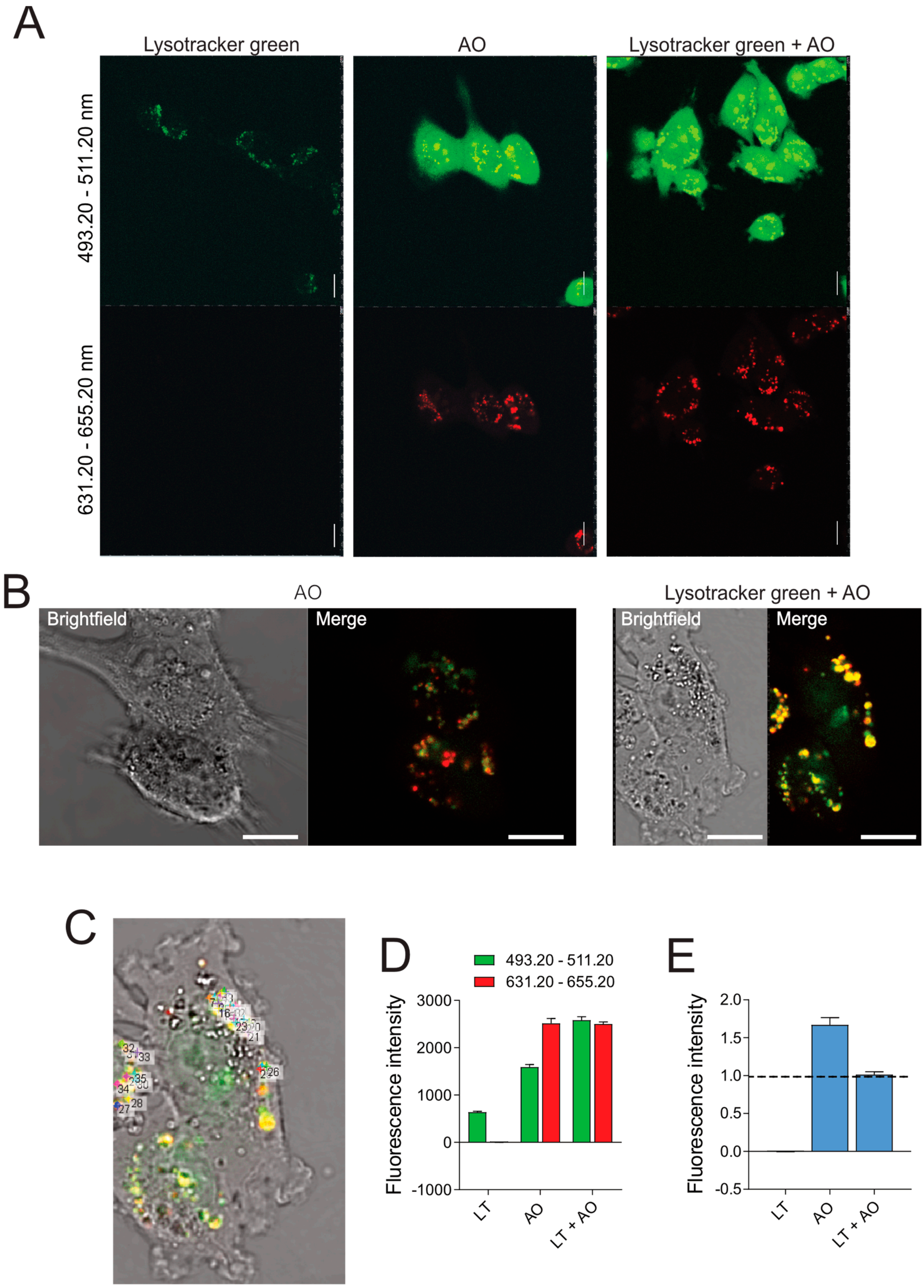

Figure S3. Co-localization of AO polymer and lysotracker in live cells. To further demonstrate that intracellular AO accumulation occurs in lysosomes, we performed a co-localization analysis between AO and LysoTracker™ Green signals in three different conditions: treated only with LysoTracker™, treated only with AO, and combined treatment. LysoTracker™ Green is a green fluorescent dye that stains specifically acidic lysosomes and that emits at 511 nm. We used spectral confocal microscopy and a virtual filter. (**A**) We collected the green signal between 493.20 and 511.20 nm for LysoTracker™, and the red signal between 631.20 and 655.20 nm for AO polymer, and the images of the two channels of a representative field for each condition are shown (scale bar 20 μm); (**B**) Merge of the green/red/brightfield channels

of AO and AO+LT conditions (scale bar 20 μm). The co-localization between Lysotracker and AO polymer in the co-treated condition was distinguishable as yellow dots in the merge field (right panel); the ratio of the red/green fluorescence intensity of several ROIs, corresponding to intracellular organelles as detected by the merge image between brightfield/green/red channels (**C**), was quantified. Both the graphs of the absolute value of the fluorescence intensity (**D**) and the ratio (**E**) are shown. In cells treated with Lysotracker alone (LT), the fluorescence intensity ratio was equal to 0 (n = 42), as expected. In cells treated with AO alone (AO), the fluorescence intensity ratio was more than 1 (median 1,499, n = 46) due to the higher red contribution of AO polymer in respect to the green contribution of AO monomer (530 nm), in acidic lysosomes. In cells treated with combined treatment (LT + AO), the fluorescence intensity ratio was almost equal to 1 (median 0.9122, n = 53) due to the presence of both high red contribution of AO polymer and green contribution of Lysotracker, in acidic lysosomes.

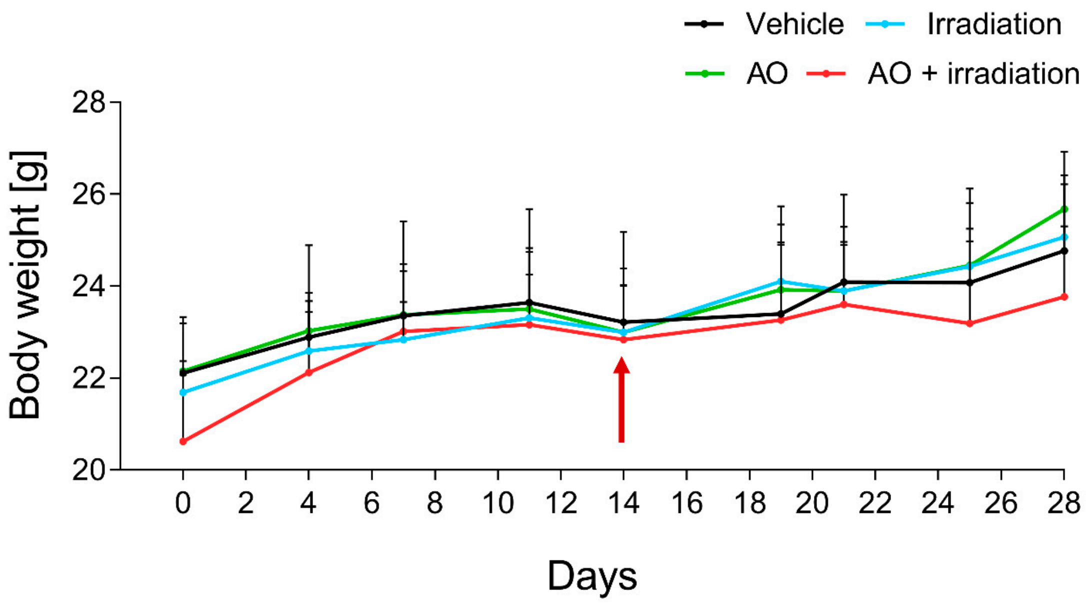

Figure S4. Mouse body weight development. The mice were weighed twice a week to monitor their clinical condition. The mean body weight (g) is presented for each study group (mean ± SEM, *n*=10 vehicle, *n*=7 irradiation, *n*=6 acridine orange, and *n*=12 acridine orange + irradiation). The red arrow indicates the day of treatment start.
